# Kangfuxin Oral Liquid Attenuates Bleomycin-Induced Pulmonary Fibrosis via the TGF-*β*1/Smad Pathway

**DOI:** 10.1155/2019/5124026

**Published:** 2019-11-03

**Authors:** Huan Yao, Shujun Wei, Yongjing Xiang, Ziqiang Wu, Weiwei Liu, Baojia Wang, Xueping Li, Huan Xu, Juan Zhao, Yongxiang Gao

**Affiliations:** ^1^College of Basic Medicine, Chengdu University of Traditional Chinese Medicine, Chengdu 11137, China; ^2^College Pharmacy, Chengdu University of Traditional Chinese Medicine, Chengdu 11137, China; ^3^College of Clinical Medicine, Chengdu University of Traditional Chinese Medicine, Chengdu 11137, China; ^4^College of International Education, Chengdu University of Traditional Chinese Medicine, Chengdu 11137, China

## Abstract

Idiopathic pulmonary fibrosis (IPF) is a fatal respiratory disease with a poor prognosis characterized by transforming growth factor (TGF)-*β*-induced proliferation, migration, and differentiation of fibroblasts, resulting in excessive extracellular matrix (ECM) deposition. Whether Kangfuxin oral liquid (KFXOL) has a protective function in pulmonary fibrosis is largely unknown. The goal of this study was to investigate the potential efficacy of KFXOL, as well as the underlying mechanism by which KFXOL regulates pulmonary fibrosis *in vivo* and *in vitro*. We found that KFXOL dramatically attenuated intratracheal bleomycin (BLM)-induced pulmonary fibrosis in terms of both severe alveolar architecture destruction and collagen deposition. KFXOL treatment significantly inhibited the proliferation, migration, and differentiation of pulmonary fibroblasts following activation using BLM/TGF-*β*1 and normalized the expression of ECM deposition-related proteins, including matrix metalloproteinase (MMP)-1, MMP-9, and tissue inhibitor of metalloproteinases 1. These effects were mediated via the inhibition of TGF-*β*1 and phosphorylated Smad2/3 activation *in vivo*. Taken together, our data suggest that KFXOL attenuates the development of pulmonary fibrosis via the TGF-*β*1/Smad signaling pathway and thus has potential utility in the treatment of pulmonary fibrosis.

## 1. Introduction

Idiopathic pulmonary fibrosis (IPF) is a chronic and progressive fibrosing interstitial pneumonia with a poor prognosis, including significant mortality [1]. IPF affects approximately 5 million individuals worldwide and is increasing in prevalence [2], although even with this high incidence, the true rate of IPF is likely higher. Current treatment options, including pirfenidone [3] and nintedanib [4], have demonstrated significant efficacy in slowing disease progression and have received approval by the United States Food and Drug Administration (FDA). However, numerous studies have concluded that these drugs cannot reverse established pulmonary fibrosis, making the long-term clinical outcomes inevitable [5–8]. Globally, IPF remains a persistent, progressive disease and the leading cause of death among respiratory diseases, with lung transplantation offering the only viable intervention in end-stage disease [1]. Thus, understanding the pathogenesis of IPF and developing novel therapeutic strategies with improved efficacy is crucial.

During IPF development, activated fibroblasts and myofibroblasts play a key role in regulating tissue repair and extracellular matrix (ECM) secretion and are a significant driver of pulmonary architecture disorders [9]. Although the underlying causes remain unknown, disease pathogenesis is characterized by an initial alveolar epithelial injury, followed by the activation and accumulation of fibroblasts via a combination of proliferation and migration to conduct tissue repair [10]. However, in IPF, this process is generally overexcited and irreversible, with fibroblasts secreting excess ECM components, leading to progressive destruction of the surrounding tissues [11]. Although multiple sources of myofibroblasts have been identified [12], the resident fibroblasts in lung tissue are considered the primary contributor of myofibroblasts.

Transforming growth factor (TGF)-*β* is an essential fibrogenic factor that regulates a variety of cellular processes including migration, proliferation, and differentiation [13]. Three major mammalian isoforms of TGF-*β* have been identified: TGF-*β*1, TGF-*β*2, and TGF-*β*3, with TGF-*β*1 being the most closely related to the development of pulmonary fibrosis [14, 15]. TGF-*β*1 content and activity are increased within the lungs of experimental fibrosis models and IPF [16, 17]. In addition, overexpression of TGF-*β*1 induces persistent pulmonary fibrosis in rodents via the canonical Smad signaling pathway. Thus, regulation of aberrant TGF-*β*1 signaling is considered an indispensable therapeutic target in IPF.

Complementary and alternative medicines have shown considerable potential in terms of both safety and efficacy for the treatment of fibrosis [18, 19]. Accordingly, these medicinal herbs and insects have received considerable interest as potential treatments for IPF. *Periplaneta americana*, also known as the American cockroach, has been used as a traditional Chinese medicine for over 2,000 years. Kangfuxin oral liquid (KFXOL), prepared as an ethanol extract of the American cockroach, is a pharmaceutical compound approved for clinical use by the China Food and Drug Administration (CFDA). Modern research has shown that American cockroach extracts exhibit a variety of biological activities including antitumor [20], gastric mucosal protection [21], wound healing [22], and immune enhancement [23]. Moreover, a recent study showed that *Periplaneta americana* extracts alleviated CCL4-induced hepatic fibrosis in rats via the inhibition of TGF-*β*1, nuclear factor kappa B (NF-*κ*B), alpha-smooth muscle actin (*α*-SMA), and tissue inhibitor of metalloproteinases 1 (TIMP-1) [24]. However, the effects of KFXOL on pulmonary fibrosis remain largely unknown.

The present study was designed to assess whether KFXOL has antifibrotic activity in an experimental animal model of IPF and to identify the potential mechanisms underlying these effects. Here, we employed a rat model of bleomycin (BLM)-induced pulmonary fibrosis to explore the antifibrotic activity of KFXOL. Furthermore, mouse lung fibroblasts were treated with TGF-*β*1 to investigate the functions of KFXOL on proliferation, migration, and differentiation. Moreover, we present *in vivo* evidence demonstrating that KFXOL suppresses the TGF-*β*1/Smad signaling pathway, suggesting a possible mechanism by which KFXOL alleviates pulmonary fibrosis.

## 2. Materials and Methods

All methods used in this study carried out in accordance with manufacturer instructions or previously published papers.

### 2.1. Chemicals and Reagents

Recombinant human TGF-*β*1 was purchased from PEPROTECH (Rocky Hill, NJ, USA). Kangfuxin oral liquid was acquired by Sichuan Good Doctor Panxi Pharmaceutical Co., Ltd. (Chengdu, Sichuan, China). BLM was purchased from Dalian Meilun Biotechnology Co., Ltd. (Dalian, Liaoning, China). Dulbecco's modified Eagle's medium (DMEM) and fetal bovine serum (FBS) were acquired from HyClone (Logan, Utah, USA). Cell Counting Kit-8 (CCK-8) was acquired from Beyotime Biotech (Shanghai, China). Primary antibody against MMP-1 (Cat. 10371-2-AP), KI67 (Cat. 27309-1-AP) and MMP-9 (Cat. 10375-2-AP) were obtained from Proteintech (Wuhan, China). Primary antibodies against Collagen-I (Cat. BIO106535) and Collagen-III (Cat. BIO10438) were acquired from BeacomBio (Birmingham, England). Primary antibodies against TIMP-1 (Cat. YT4658) and *α*-SMA (Cat. YT5797) were acquired from ImmunoWay (Plano, TX, USA). Primary antibody against phospho-Smad2/3 (Cat. WL02305) was acquired from Wanleibio (Shenyang, China). Avidin/Biotin Blocking Kit, ABC HRP Kit, Biotinylated Rabbit Anti-Rat IgG antibody and DAB Substrate Kit were acquired from Vector Labs (Burlingamge, CA, USA).

### 2.2. Animals

Adult male Sprague-Dawley rats (180–200 g) were purchased from Chengdu Dashuo Experimental Animal Center. The animals were acclimatized in a clean layer flowing room with a temperature of 20 ± 2°C, relative humidity (RH) 60 ± 15%, 12/12 h light/dark cycle and allowed ad libitum feeding. All the animal experiments in the study were approved by the Experimental Animal Ethics Committee at Chengdu University of Traditional Chinese Medicine in accordance with NIH guidelines.

### 2.3. Animal Treatment

All the animals were randomly divided into one of the six groups, 8 rats in each group: (1) saline intratracheally plus saline intragastrically (normal control group, Normal, *n* = 8), (2) intratracheal BLM plus saline intragastrically (model group, BLM, *n* = 8), (3) intratracheal BLM plus 10 mL/kg Kangfuxin (high-dose group, HDKFX, *n* = 8), (4) intratracheal BLM plus 5 mL/kg Kangfuxin (medium-dose group, MDKFX, *n* = 8), (5) intratracheal BLM plus 2.5 mL/kg Kangfuxin (low-dose group, LDKFX, *n* = 8) [25], and (6) intratracheal BLM plus 3 mg/kg dexamethasone (positive control group, DXM, *n* = 8). A nonsurgical rat model of pulmonary fibrosis was established as previously described [26]. Briefly, BLM (5.0 mg/kg) [27] in saline or saline alone was administered intratracheally to rats on day 0. One day after BLM treatment, saline or Kangfuxin or dexamethasone was administered orally daily for 21 days [28]. Immediately after all the rats were euthanized, lungs were harvested for the following experiments.

### 2.4. Pulmonary Index

The pulmonary index (PI) refers to the ratio of lung wet to body mass. PI = rat lung wet weight (g)/rat body mass (kg) × 100%.

### 2.5. Histopathological Examination

Upper lobes of the right lung were fixed in 4% paraformaldehyde in 0.1 M PBS overnight and embedded in paraffin; 5-*μ*m thickness of slides was collected and deparaffinized. Hematoxylin/eosin (H&E) staining and Masson's trichrome staining were performed as previously described [29].

### 2.6. Ashcroft Scale

Semiquantification of lung fibrosis in histological lung sections was done using the modified Ashcroft scale as previously described [30].

### 2.7. Immunohistochemistry

Lung tissue sections were deparaffinized and rehydrated using a graded ethanol series. After antigen retrieval in citric acid at 98°C for 10–15 minutes, eliminating endogenous peroxidase and pre-incubating with 5% BSA to block background staining, sections were incubated with anti-KI67 antibody (1 : 150), anti-*α*-SMA antibody (1 : 200), anti-MMP-1 antibody (1 : 150), anti-MMP-9 antibody (1 : 200), anti-TIMP-1 antibody (1 : 200), anti-Collagen I antibody (1 : 200), anti-Collagen III antibody (1 : 200), or anti-Smad2/3 antibody (1 : 200) at 4°C overnight. Followed by incubation with biotinylated secondary antibody at room temperature for 1 hour (1 : 200) and ABC solution for 30 minutes at room temperature. The expression of targets visualized by DAB solution and counterstained with hematoxylin.

### 2.8. Cell Culture

Cells were cultured in DMEM supplemented with 10% FBS and antibiotics (100 KU/L penicillin and 100 mg/L streptomycin) in an incubator at 37°C with 5% CO_2_ atmosphere.

### 2.9. Cell Preparation

Lung fibroblasts from C57BL/6 mouse (MLFs) were obtained according to a protocol previously established [31]. Briefly, the mice were euthanized, and the chest area was washed with 70% ethanol. The mouse chest was cut with sterile forceps and scissors; lung tissues were collected and transferred to a tissue culture dish and cut into small pieces. The tissue fragments were transferred to a sterile centrifuge tube, and cell digestive solution (containing 5% trypsin and type II collagenase) was added and incubated at 37°C for 3–5 minutes. After that, the supernatant was transferred to a new centrifuge tube, and the digestion was terminated by the addition of DMEM medium containing 10% FBS. The remaining lung tissue fragments were repeatedly digested until intact lung tissue fragments were not observed. Collecting the digestive solution and centrifuged at 524 g for 5 minutes, the supernatant was removed, and the cell pellet was washed 3 times with DMEM. After that, the cell pellet was resuspended with DMEM containing 10% FBS and transferred to a tissue culture dish and placed in a tissue culture incubator at 37°C, 5% CO_2_. After 30 minutes, the medium was removed, and the culture medium was added again. The above procedure was repeated at the time of cell passage, and when passaged to the fourth generation, subsequent experiments were performed.

### 2.10. Cell Viability Assay

3 × 10^3^ MLFs (each well) were seeded in a 96-well culture plate and treated with different concentrations of Kangfuxin oral liquid in the presence and absence of TGF-*β*1 (10 ng/mL) as indicated for 48 h. Cell viability was measured using according to the manufacturer' s instructions of Cell Counting Kit-8.

### 2.11. Cell Counting

5 × 10^4^ MLFs (each well) were seeded in a 6-well culture plate and treated with Kangfuxin oral liquid in the presence and absence of TGF-*β*1 (10 ng/mL). Then, the fragments were washed with PBS, trypsinized and the cell numbers counted by automated Cell Counter IC1000 System (Countsatar, USA) at 12 h, 24 h, 36 h, and 48 h.

### 2.12. Transwell Migration Assay

Cultured, enriched MLFs were resuspended at 1 × 10^6^ cells/mL in DMEM containing 10% FBS. Medium alone (negative control) or medium containing Kangfuxin oral liquid in the presence and absence of TGF-*β*1 (10 ng/mL) (total 600 *μ*L) was added to individual wells of a 24-well plate. Transwell devices then were inserted, and the fibroblasts (100 *μ*L) were layered on top of the membrane. After 24 h treatments, the number of transferred cells monitored after crystal violet staining.

### 2.13. Quantitative Real-Time PCR

Total RNA was extracted from lung tissues and cells using TRIzol^TM^ Reagent (Invitrogen, USA) according to the manufacturer's instruction, and qRT-PCR was done as described previously [32]. Briefly, a 0.5–1 *μ*g sample of total RNA was used for first-strand cDNA synthesis using iScript™ cDNA Synthesis Kit (Bio-Rad, USA). Then the mRNA level of cyclin D1, p15ink4b, p19arf, p18in4c, and TGF-*β*1 were analyzed by quantitative real-time PCR (Bio-Rad, USA) using iScript™ one-step RT-PCR Kit with SYBR® Green (Bio-Rad, USA) in a total volume of 20 *μ*L with gene-specific primers listed in table (Supplementary [Supplementary-material supplementary-material-1]).

### 2.14. Data Analysis

Quantitative data were expressed as mean ± standard deviation (SD); statistical analysis was performed using Students *t*-test or One-way analysis of variance. A value of *P* < 0.05 was considered statistically significant.

## 3. Results and Discussion

### 3.1. KFXOL Alleviates BLM-Induced Pulmonary Fibrosis in Rats

**Figure 1:**
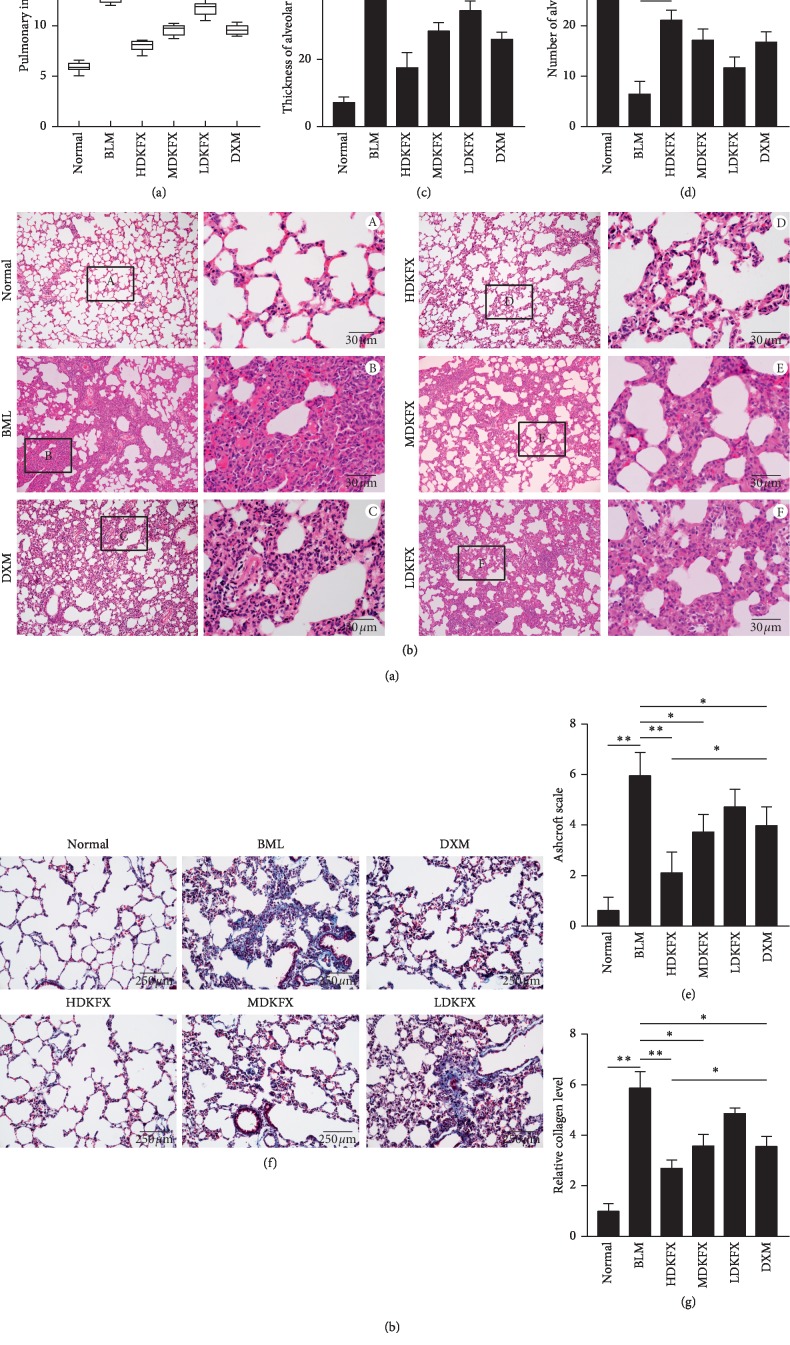
KFXOL alleviates bleomycin (BLM)-induced pulmonary fibrosis in rats. (a) Pulmonary indexes were compared among different treatment groups 21 days after BLM administration. (b) Representative images of H&E staining in lung tissues from different groups; the thickness and number of alveoli differed significantly in (c) and (d). (e) Ashcroft scales of different groups were analyzed based on images of H&E staining. (f) Lung tissue sections were prepared and stained with Masson's trichrome staining, and the relative collagen level of each group was quantified in (g) using ImageJ software. Data are presented as the mean ± SD; *n* = 8; ^*∗*^*P* < 0.05; ^*∗∗*^*P* < 0.01.

To assess the antifibrotic potential of KFXOL *in vivo*, we relied on the murine model of BLM-induced pulmonary fibrosis. In this model, rats were treated with BLM (5.0 mg/kg) and Kangfuxin (i.g.) as indicated for 21 consecutive days. Intratracheal injection of BLM decreased the body weight and increased the wet lung weight in rats, leading to significant increases in pulmonary index (IP) scores. Treatment with KFXOL, particularly high-dose treatments, dramatically reduced IP levels in rats (Figure 1(a)). To verify the effects of KFXOL on lung fibrosis, we performed hematoxylin and eosin (H&E) staining on sections of paraffin-embedded rat lung. BLM significantly induced pulmonary fibrosis in rats, as evidenced by the increased alveolar thickness and decreased alveolar numbers. Surprisingly, KFXOL treatment inhibited BLM-induced damage in a dose-dependent manner to levels similar to those of normal lung architecture (Figures 1(b)–1(e)). Similarly, KFXOL significantly reduced excessive deposition of collagen fibers due to BLM, as determined by Masson's trichrome staining (Figures 1(f) and 1(g)). Collectively, these data support the potential value of KFXOL for the treatment of pulmonary fibrosis.

### 3.2. KFXOL Suppresses the Proliferation and Differentiation of Mouse Lung Fibroblasts *In Vitro* and *In Vivo*

**Figure 2:**
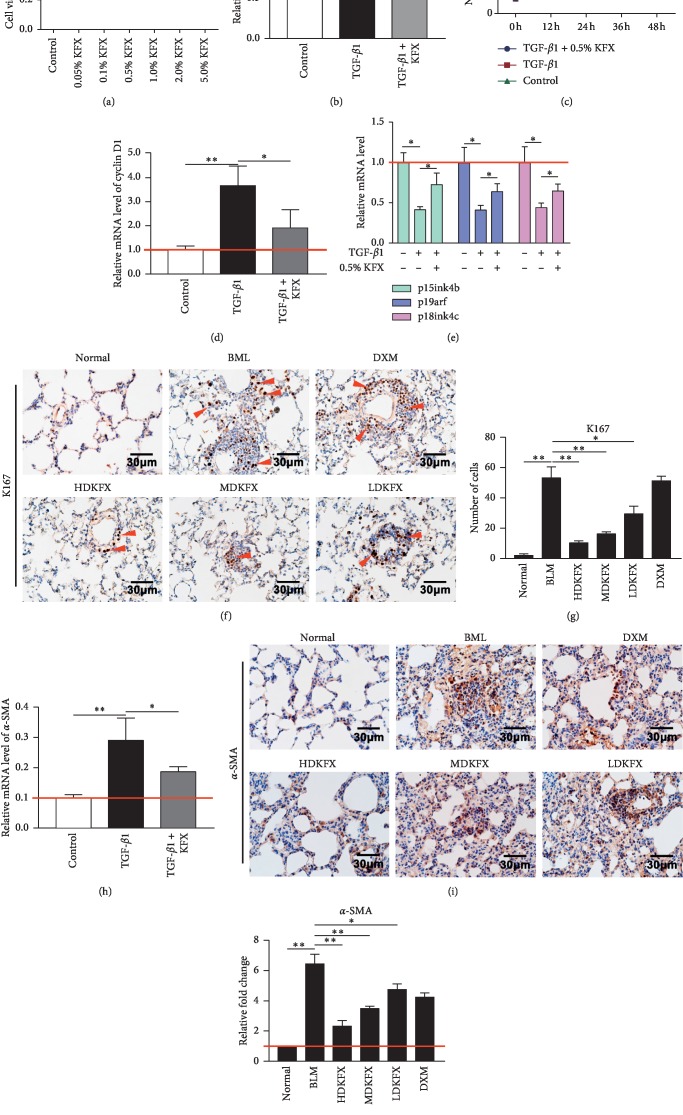
KFXOL suppresses the proliferation and differentiation of mouse lung fibroblasts (MLFs) *in vitro* and *in vivo*. (a) A CCK-8 assay was performed to assess cell viability after treatment with various concentrations of KFXOL for 48 h. (b) The relative viabilities of MLFs were measured by CCK-8 assay at 48 h Values were normalized to GF-*β*1-treated controls, representing 100% cell viability. (c) The growth of MLFs was accomplished by cell counting at different time points. (d, e) Quantitative real-time PCR (qPCR) analysis was used to assess the expression of cell cycle positive and negative regulated genes. (f) Representative IHC images stained using anti-KI67 antibodies; staining intensities are quantified in (g). (h) qPCR analysis of *α*-SMA expression *in vitro*. (i) Representative IHC staining of *α*-SMA in lung tissues and the relative expression levels of *α*-SMA were quantified in (j) using ImageJ software. Data are presented as the mean ± SD, with all experiments performed in triplicate; *n* = 8; ^*∗*^*P* < 0.05; ^*∗∗*^*P* < 0.01.

Next, we examined the potential mechanisms underlying the antifibrotic effects of KFXOL. The pathogenesis of pulmonary fibrosis is caused by excessive proliferation of active fibroblasts in response to TGF-*β*1 following alveolar epithelial cell injury. Here, we used mouse lung fibroblasts (MLFs) as a model to investigate the effects of KFXOL on the proliferation of fibroblasts and active fibroblasts. MLFs were treated with media containing a gradient of KFXOL *in vitro* for 48 h to identify the optimal concentration of KFXOL. Samples were then examined using a CCK-8 assay to assess the effects on cell viability. Our data indicated that KFXOL inhibited cell viability in a concentration-dependent manner, with 0.5% KFXOL identified as the appropriate concentration for cell treatment (Figure 2(a)). Thus, we selected 0.5% KFXOL for cell treatment in the subsequent experiments.

Treatment of MLFs with TGF-*β*1 led to an increased number of viable fibroblasts; addition of 0.5% KFXOL led to a reduction in cell viability, as well as a decrease in overall cell numbers relative to TGF-*β*1-treated controls (Figures 2(b) and 2(c)). Further analyses were performed using quantitative real-time PCR (qPCR) to investigate the expression of proliferation-associated genes as well as negative regulators of the cell cycle, including cyclin D1, p15ink4b, p19arf, and p18ink4c. KFXOL treatment attenuated changes in cyclin D1, p15ink4b, p19arf, and p18ink4c expression relative to TGF-*β*1-treated controls (Figures 2(d) and 2(e)).

Next, we performed immunohistochemical (IHC) staining of rat lung tissues for KI67 to examine the effects of KFXOL on cell proliferation *in vivo*. Impressively, KFXOL treatment significantly attenuated BLM-induced expression of KI67 in rat lung tissue in a dose-dependent manner (Figures 2(f) and 2(g)).

Fibroblasts are activated and differentiated into myofibroblasts, specialized contractile cells with higher profibrotic potential than fibroblasts [33]. To further validate the effects of KFXOL on the differentiation of activated fibroblasts *in vitro* and *in vivo*, we used qPCR to investigate the expression of *α*-SMA in fibroblasts treated with KFXOL for 36 h. Results were then confirmed by IHC using slides of rat lung tissue to detect the expression of *α*-SMA. qPCR results indicated that KFXOL suppressed the expression of *α*-SMA *in vitro* relative to TGF-*β*1-treated controls (Figure 2(h)). Similarly, IHC staining indicated that BLM was sufficient to induce enrichment of *α*-SMA in lung tissue. These effects were dramatically attenuated following treatment with KFXOL, in a dose-dependent manner (Figures 2(i) and 2(j)). Taken together, these data demonstrate that KFXOL inhibited the proliferation of MLFs via the attenuation of cell cycle gene expression and myofibroblast differentiation.

### 3.3. KFXOL Suppresses MLF Migration and Attenuates Imbalances in ECM Degradation *In Vivo*

**Figure 3:**
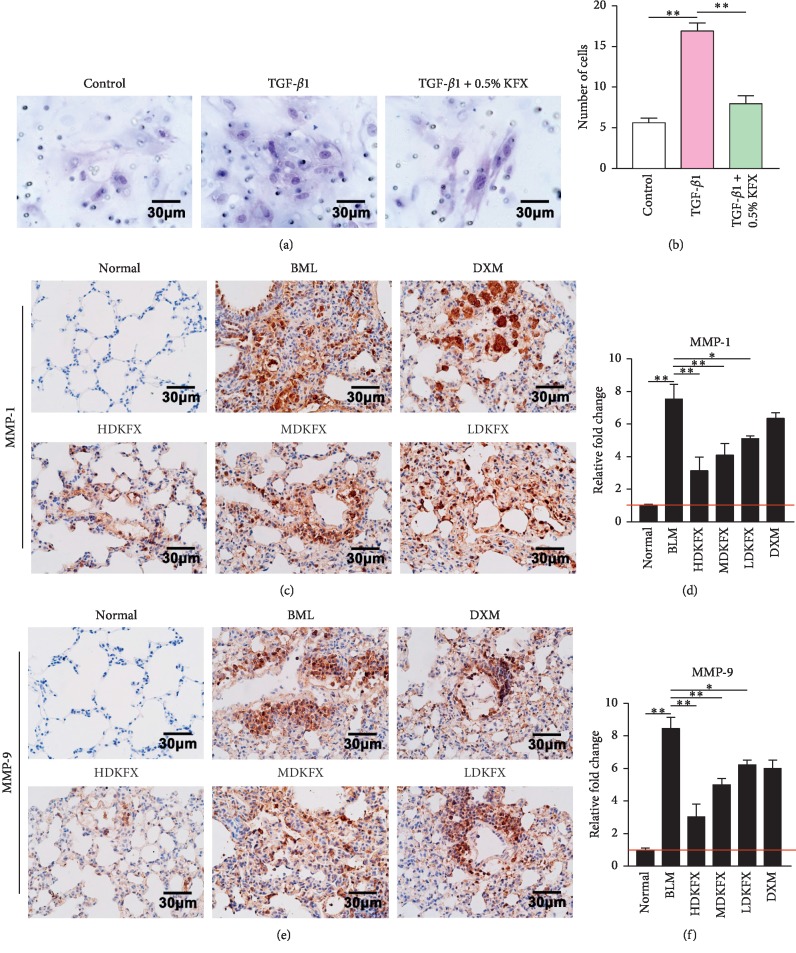
KFXOL suppresses MLF migration and improves the imbalance of ECM degradation *in vivo*. (a) A transwell assay was used to assess the effect of KFXOL on fibroblast migration. Assay results are quantified in (b). Data are presented as the mean ± SD (^*∗∗*^*P* < 0.01), with each experiment performed in triplicate. (c) Representative IHC staining of MMP-1 in lung tissue and the expression levels of MMP-1 were quantified in (d). (e) Representative IHC staining of MMP-9 in lung tissues and the expression levels of MMP-9 were quantified in (f) using ImageJ software. Data are expressed as the mean ± SD; *n* = 8; ^*∗*^*P* < 0.05, ^*∗∗*^*P* < 0.01.

Migration of activated fibroblasts contributes to the formation of fibroblastic foci and is associated with the degradation of ECM, whereas matrix metalloproteinases (MMP) exhibit the opposite effect, serving as essential regulators of ECM degradation. Accordingly, we examined the effects of KFXOL on the migration of activated fibroblasts, and whether it acts as a negative regulator of BLM-induced activation of MMPs. A transwell assay was used to investigate the potential inhibitory effects of KFXOL on activated fibroblast migration, revealing significant increases in transferred MLFs following treatment with TGF-*β*1. These effects were significantly attenuated in wells treated with KFXOL (Figures 3(a) and 3(b)). Next, we used IHC to assess the expression of MMP-1 and MMP-9 protein in mouse lung tissue, revealing marked increases in protein expression in BLM-treated mice. Treatment with KFXOL significantly decreased MMP-1 and MMP-9 expression in BLM mice in a dose-dependent manner (Figures 3(c)–3(f)).

### 3.4. KFXOL Reduces Collagen Production *In Vitro* and *In Vivo*

**Figure 4:**
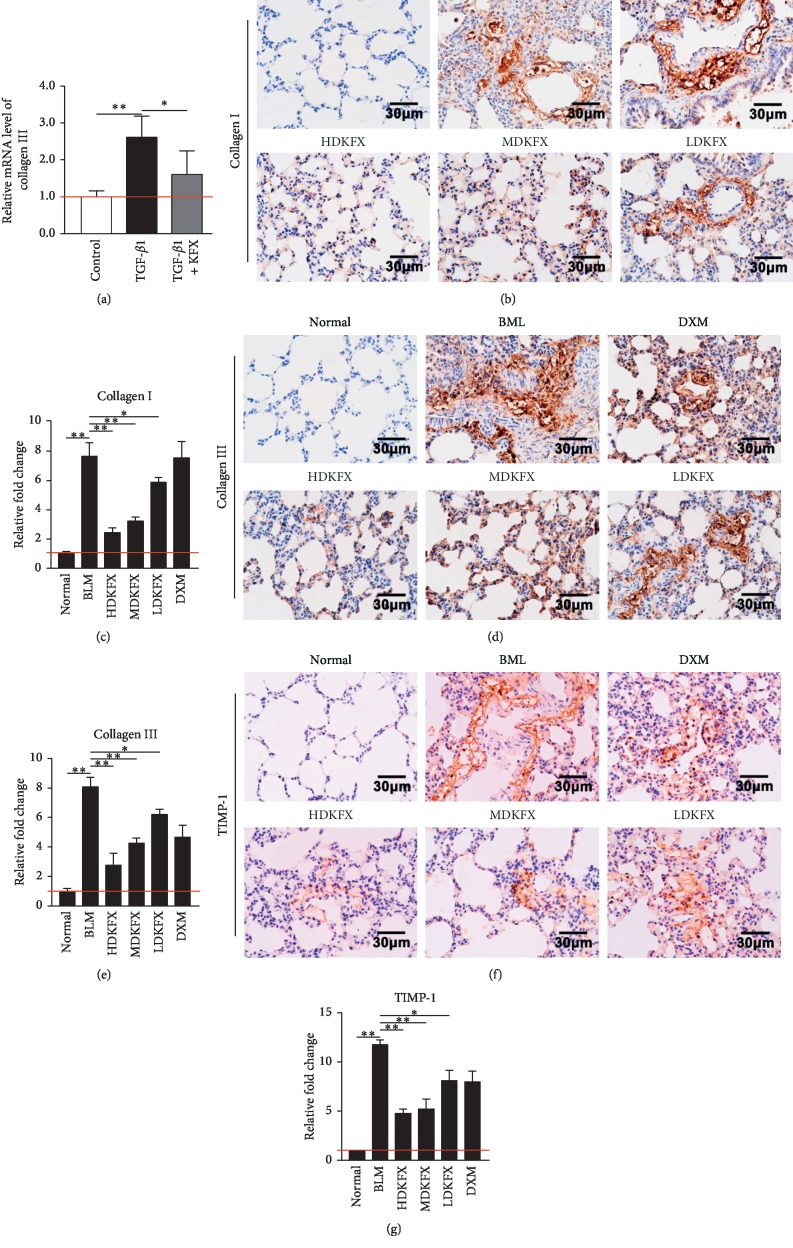
KFXOL reduces collagen production *in vitro* and *in vivo*. (a) qPCR analysis of TGF-*β*1 expression in fibroblasts. Data are presented as the mean ± SD; all comparisons were performed in triplicate; ^*∗*^*P* < 0.05; ^*∗∗*^*P* < 0.01. (b) Representative IHC staining of collagen I in lung tissues; collagen I expression is quantified in (c). (d) Representative IHC staining of collagen III in lung tissues; collagen III expression is quantified in (e). (f) Representative IHC staining of TIMP-1 in lung tissues; TIMP-1 expression is quantified in (g). Data were quantified using ImageJ software and presented as the mean ± SD; *n* = 8; ^*∗*^*P* < 0.05; ^*∗∗*^*P* < 0.01.

Excessive deposition of collagen in lung tissue results in severe pulmonary defects via the disruption of alveolar function, highlighting the critical role of collagen production during pulmonary fibrosis development. qPCR was used to determine whether KFXOL is effective for suppression of collagen production. As shown in Figure 4(a), TGF-*β*1-treated cells exhibited increased expression of collagen III relative to controls; these effects were significantly attenuated following treatment with KFXOL.

Next, we examined levels of both collagen I and collagen III on lung tissue slides using IHC. IHC results showed that BLM promoted the expression of collagen I and collagen III, which was reduced by KFXOL treatment in a dose-dependent manner (Figures 4(b)–4(e)).

Based on these observations, we next investigated the mechanisms underlying the effects of KFXOL on collagen deposition. TIMP-1 was previously identified as a negative regulator of collagen degradation and an important contributor to collagen accumulation [34]. To assess the involvement of TIMP-1, we performed IHC using an anti-TIMP-1 antibody to identify the efficacy of KFXOL in regulating TIMP-1 expression under BLM stimulation. We found that KFXOL downregulated the expression of TIMP-1 following abnormal increases in BLM rats (Figures 4(f) and 4(g)). Collectively, these data show that KFXOL reduces collagen production in rat lung tissue, thereby attenuating the progression of pulmonary fibrosis.

### 3.5. KFXOL Inhibits TGF-*β*1/Smad Pathway *In Vivo*

**Figure 5:**
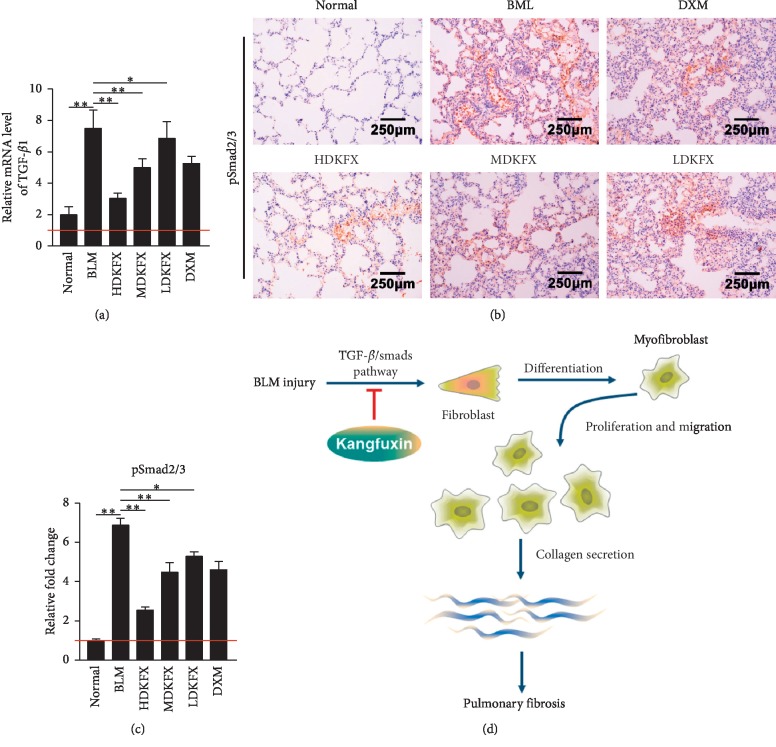
KFXOL inhibits the TGF-*β*1/Smad pathway *in vitro* and *in vivo*. (a) qPCR analysis of TGF-*β*1 expression in rats. Data are presented as the mean ± SD, with each group performed in triplicate; ^*∗*^*P* < 0.05; ^*∗∗*^*P* < 0.01. (b) IHC was used to assess the expression of phosphorylated Smad2/3 in lung tissues; the relative expressions of these proteins are quantified in (c) using ImageJ software. Data are presented as the mean ± SD; *n* = 8; ^*∗*^*P* < 0.05; ^*∗∗*^*P* < 0.01. (d) The schematic diagram demonstrates the functional role of KFXOL in attenuating pulmonary fibrosis in a murine model induced by BLM intratracheal administration via the TGF-*β*1/Smad signaling pathway.

Next, we aimed to understand how KFXOL suppresses fibroblast activation. Recent studies showed that the canonical TGF-*β*/Smad signal pathway serves as a regulator of fibroblast activity, with Smad2 and Smad3 serving as the two major downstream regulators that promote TGF-*β*1-mediated tissue fibrosis [35]. Therefore, we wondered whether BLM-induced changes in TGF-*β*1/Smad signaling pathway activation may be regulated by KFXOL. To determine the effects of KFXOL on pathway activation, qPCR was used to examine TGF-*β*1 expression in rats. Our data showed that KFXOL treatment significantly downregulated TGF-*β*1 expression in a dose-dependent manner, relative to BLM-treated controls (Figure 5(a)). Further analyses showed that pSmad2/3 were upregulated upon BLM simulation and that these effects were attenuated in a dose-dependent manner following KFXOL treatment (Figures 5(b) and 5(c)). Collectively, these data indicate that KFXOL alleviates BLM-induced pulmonary fibrosis via the TGF-*β*1/Smad signaling pathway.

## 4. Conclusions

In this study, we provide evidence that KFXOL has significant therapeutic potential for the treatment of pulmonary fibrosis. KFXOL suppressed TGF-*β*1-induced fibroblast proliferation via regulation of the cell cycle. KFXOL was further shown to inhibit fibroblast migration, further contributing to the inhibition of fibroblast accumulation. In addition, KFXOL significantly downregulated the expression of collagen fibers in rats administered BLM. We also demonstrated that KFXOL antagonized TGF-*β*1-mediated canonical Smad signaling, revealing a potential mechanism by which KFXOL attenuates pulmonary fibrosis (Figure 5(d)).

IPF is a severe respiratory disease with tremendous mortality and morbidity. The use of animal models has proven instrumental to the current understanding of disease pathogenesis, as have many of the animal models used to study the pathophysiology of lung diseases. Although several methods have been established, the standard reagent commonly used to induce experimental pulmonary fibrosis in animals is BLM [36]. BLM is an antitumor chemotherapy drug produced by the bacterium *Streptomyces verticillus*, which has the side effects of cytotoxicity, with the strongest effects seen in the lung [36]. Intratracheal exposure is the standard route of BLM administration for pulmonary fibrosis models. Previous studies have shown that BLM induces considerable biochemical and histological defects, which contributes to pulmonary fibrosis. Thus, a BLM-induced rat model of pulmonary fibrosis was selected in our study to elucidate the effects of KFXOL and to identify the mechanism underlying its antifibrotic effects.

Several common methods have been developed to assess fibrosis, including the pulmonary index, a semiquantitative histological analysis based on the scoring system by Ashcroft [30], and collagen content. The pulmonary index is a general indicator of pulmonary fibrosis, which is mainly determined based on the wet weight of the lung. In fibrotic lung tissue, significant increases in the pulmonary index are typically observed due to overexpression of collagen fibers. Interestingly, we found that KFXOL dramatically attenuated pulmonary index scores caused by BLM, in a dose-dependent manner. Histopathological impairment is an important indicator of IPF diagnosis and disease prognosis. We therefore performed H&E staining and Masson's trichrome staining of rat lung sections to observe the histological structure of the lung, after which we utilized the Ashcroft score to assess the degree of fibrosis. Our data showed that KFXOL significantly improved histopathological disorders such as fibrous mass formation and collagen deposition. Importantly, no side effects were observed in our study, consistent with previous reports, indicating that KFXOL has a favorable safety profile *in vivo*. Based on the above findings, we believe that KFXOL has a protective activity in BLM-induced pulmonary fibrosis in rats and may be used clinically as an antifibrotic therapy.

Myofibroblast production is another phenotype triggered by TGF-*β*1 activation of fibroblasts, characterized by excessive expression of *α*-SMA [37]. In normal acute wounds, myofibroblasts are transiently present and orchestrate time-limited and spatially restricted scar formation. However, when myofibroblasts persist at the site of an injury, it leads to excessive deposition of ECM. IPF is characterized by abnormal increases in fibroblasts, as well as their differentiated phenotype, myofibroblasts [33].

Cell proliferation is a well-characterized process regulated by cell cycle factors, including cyclins and cyclin-dependent kinases (CDK), as well as CDK inhibitors (CKIs) [38]. We further sought to investigate the extent to which KFXOL inhibits the proliferation and differentiation of both fibroblasts and myofibroblasts. Our data indicate that KFXOL treatment reduced the viability of mouse lung fibroblasts, resulting in subdued proliferation at different time points. Further analyses suggested that this is associated with a return to normal CKI and cyclin expression (Figures 2(d) and 2(e)). Impressively, KFXOL significantly repressed upregulation of *α*-SMA in BLM-treated rats and TGF-*β*1-treated MLFs (Figures 2(h)–2(j)).

Previous studies have shown that a number of MMPs regulate processes implicated in IPF pathogenesis and promote pulmonary fibrotic responses to injury [39]. In addition, MMPs induce ECM degradation and promote fibrocyte migration [40]. We further investigated the effects of KFXOL on cellular migration in fibroblasts and activated fibroblasts, as well as the expression of MMPs in lung sections. Our data demonstrated that KFXOL markedly inhibited MLF migration. Consistent with this finding, KFXOL also attenuated the expression of MMP-1 and MMP-9 (Figure 3).

TIMPs are negative regulators of ECM degradation, serving as crucial regulators of MMP activity [41, 42]. The role of TIMPs in IPF has been identified [39]. TIMP-1, a prototypic and original ancestral member of the TIMP family, is the most widely distributed and acts on all active MMPs. TIMP-1 has also been shown to participate in ECM accumulation by preventing MMP-induced ECM degradation [39]. Our data indicate that KFXOL dramatically inhibited TIMP-1 expression, which is consistent with previous observations on the activity of *P*. *americana* extracts in rats with CCL4-induced hepatic fibrosis [24]. Moreover, KFXOL also reduced the levels of type I and type III collagens, two primary components of ECM, suggesting that KFXOL maintained the balance between synthesis and degradation of ECM components to improve pulmonary architecture (Figure 4).

In this study, we also investigated the potential mechanisms by which KFXOL attenuates pulmonary fibrosis. TGF-*β*1 exerts multiple biological functions, including pulmonary inflammation, cell apoptosis, and cell proliferation, as well as fibrosis. TGF-*β*1 functions through a variety of Smad-independent pathways, including p38, extracellular signal-regulated kinase (ERK), and mitogen-activated protein kinase (MAPK), as well as the canonical Smad-dependent pathway [43, 44]. In this study, we focused on the pathological roles of TGF-*β*/Smad signaling. The TGF-*β*/Smad signaling pathway has been shown to play a critical role in pulmonary fibrosis. TGF-*β*1 binds TGF-*β*RII, leading to activation of TGF-*β*RI. This signaling cascade leads to the phosphorylation of Smad2 and Smad3, which serve as central mediators of TGF-*β*/Smad signaling [44]. Surprisingly, our results showed that KFXOL significantly blocked both TGF-*β*1 and pSmad2/3 expression, both of which were overexpressed in rats with BLM-induced pulmonary fibrosis (Figure 5).

Interestingly, our *in vivo* study also demonstrated that dexamethasone treatment suppressed pulmonary fibrosis, likely due to its anti-inflammatory effects. Although KFXOL was superior to dexamethasone in terms of its antifibrotic effects, traditional therapies such as glucocorticoids and immunosuppressive agents have proven to be ineffective in clinical trials [45], suggesting a difference in the pathogenesis of animal models relative to human IPF. Further studies are therefore necessary to fully assess the efficacy of KFXOL for the treatment of IPF.

Type II alveolar epithelial cells are thought to function as stem cells in the lungs. Recent studies reported that alveolar epithelial cells can acquire a mesenchymal phenotype via the epithelial-mesenchymal transition (EMT) in the process of IPF development [46, 47]. Drugs targeting EMT may have promise as potential therapies for the treatment of pulmonary fibrosis. Surprisingly, a large number of compounds that are synthesized or derived from natural products suppress EMT by targeting some of the primary mediators of fibrosis [48]. However, the mechanisms of KFXOL on EMT remain poorly understood, and whether KFXOL inhibits pulmonary fibrosis via the EMT pathway remains to be elucidated.

In summary, our results indicate that KFXOL inhibits the TGF-*β*/Smad signaling pathway, decreasing the differentiation, proliferation, and migration of fibroblasts, and decreasing ECM deposition, leading to an improvement in BLM-induced pulmonary fibrosis.

## Data Availability

All data included in this study are available upon request by contact with the corresponding author.

## Supplementary Materials

Supplementary Materials Supplementary Table 1: primer sets used for real-time PCR.
