# Supplementary material for: Kangfuxin Oral Liquid Attenuates Bleomycin-Induced Pulmonary Fibrosis via the TGF-β1/Smad Pathway
Source: Evid Based Complement Alternat Med. 2019 Nov 3;2019:5124026. doi: 10.1155/2019/5124026 (PMC6926420; doi:10.1155/2019/5124026)
Supplement: Supplementary Materials — Supplementary Table 1: primer sets used for real-time PCR. [file 5124026.f1.pdf]

Supplementary Table 1. Primer sets used for real-time PCR.

Gene	Sense Primer (5′ - 3′)	Antisense Primer (5′ - 3′)
Cyclin D1 (Mouse)	GCGTACCCTGACACCAATCTC	ACTTGAAGTAAGATACGGAGGGC
p15ink4b (Mouse)	ATGTTGGGCGGCAGCAGTGACG	ATCTCCAGTGGCAGCGTGCAG
p18ink4c (Mouse)	GATTTGGGAGAACTGCGCTG	GCCTGGAACTCCAGCAAAGC
α-SMA (Mouse)	AGAACACGGCATCATCAC	TCCAGAGTCCAGCACAAT
Collagen-III (Mouse)	TCCTGAAGATGTCGTTGATGTG	GTCTCATTGCCTTGCGTGTT
p19afr (Mouse)	GGGTCGCAGGTTCTTGGTC	GTGCGGCCCTCTTCTCAA
TGF-β1 (Rat)	ACCGCAACAACGCAATCTA	AATGGGGGTTCTGGCACT
GAPDH (Mouse)	CATGGCCTTCCGTGTTCCTA	GCCTGCTTCACCACCTTCTT
GAPDH (Rat)	GGCAAGTTCAACGGCACA	TCTCGCTCCTGGAAGATGG
